# Lutte contre la mortalité maternelle en milieu rural: décentralisation de l’offre des soins obstétricaux d’urgence au Burkina Faso

**DOI:** 10.11604/pamj.2017.27.236.12952

**Published:** 2017-08-01

**Authors:** Souleymane Kaboré, Clément Ziemlé Méda, Issiaka Sombié, Léon Blaise Savadogo, Robert Karama, Koabié Bakouan, Djénéba Sanon Ouédraogo, Norbert Coulibaly, Robert Lucien Kargougou, Emanuel Lankoandé, Ramatou windsouri Sawadogo, Karen Gosch

**Affiliations:** 1 Institut Supérieur des Sciences de la Santé, Université de Bobo-Dioulasso/Burkina Faso; 2 Direction Régionale de la Santé de la Boucle du Mouhoun/Burkina Faso; 3 Organisation Ouest Africaine de la Santé; 4 Fonds des Nations Unies pour la Population Burkina; 5 Fonds des Nations Unies pour la Population Sénégal; 6 Secrétariat Général du Ministère de la santé du Burkina Faso; 7 Direction de la Santé de la Famille du Burkina Faso; 8 Deutsche Gesellschaft für Internationale Zusammenarbeit (GIZ)

**Keywords:** Complication obstétricale, décentralisation, décès maternel, ressource humaine, sage-femme de zone, Obstetrical complication, decentralization, maternal deaths, human resource, zonal midwife

## Abstract

**Introduction:**

Pour combler la pénurie en sages-femmes (SF) dans le district sanitaire de Tougan au Burkina Faso, il a été conçu une stratégie de décentralisation de l'offre des soins obstétricaux d'urgence basée sur des interventions ponctuelles de prise en charge des complications obstétricales au niveau des centres de santé (CS) en milieu rural par des SF. La présente étude a eu pour objectif de décrire cette expérience et d'analyser ses résultats.

**Méthodes:**

Il s'agit d'une étude intervention de type transversal analytique basée sur une revue des données routinières de l'ensemble des parturientes prises en charge de 2013 à 2015. La collecte des données s'est déroulée du 5 au 20 janvier 2016. Un test Chi^2^, des rapports de cotes (OR) et leurs intervalles de confiance à 95% ont été calculés.

**Résultats:**

Au total 416 parturientes présentant des complications obstétricales ont été prises en charge par les SF de zone. L'âge moyen des parturientes était de 26.4 ans. La distance médiane parcourue pour prendre en charge les parturientes était de quinze km pour un délai moyen d'intervention de 21.1 minutes (déviation standard = 7.13 minutes). Les dystocies représentaient la moitié (50.7%, IC95% = 45.8-55.6) des complications prises en charge suivies des hémorragies (26.4%, IC95% = 22.3%-31.0%). Plus de 77% des interventions avaient abouti à la résolution locale des complications obstétricales. Enfin, le résultat de l'intervention était fonction de la pathologie prise en charge (OR = 5.88; p < 0.001).

**Conclusion:**

Cette stratégie a permis d'apporter une réplique à l'absence de SF dans les CS périphériques du district sanitaire de Tougan. Dans ce contexte particulier, cette intervention pourrait apporter une solution alternative au manque de ressources humaines en santé en milieu rural.

## Abstract (en)

**Introduction:**

In order to cover the shortage of midwives (MWs) in the health district of Tougan, Burkina Faso, a strategy for the decentralization of emergency obstetric cares, based on ad hoc interventions undertaken by MW to manage obstetric complications in health centers (HCs), has been developed in rural areas. This study aimed to describe this experience and to analyze the achieved results.

**Methods:**

We conducted a cross-sectional, analytical, intervention study based on a review of routine data from all the parturients treated from 2013 to 2015. Data collection took place from 5 to 20 January 2016. Chi-square Test, odds ratios (ORs) and their 95% confidence intervals were calculated.

**Results:**

A total of 416 parturients with obstetric complications were treated by zonal MW. The average age of patients was 26.4 years. The median distance travelled to treat parturients was 15 km, with an average intervention period of 21.1 minutes (standard deviation = 7.13 minutes). Dystocias accounted for half (50.7%, CI95%= 45.8-55.6) of treated complications followed by hemorrhage (26.4%, CI95%= 22.3%-31.0%). More than 77% of interventions resulted in local resolution of obstetric complications. Finally, the intervention outcome was subject to the pathology treated (OR=5.88; p < 0.001).

**Conclusion:**

This strategy was an answer to the shortage of MWs in the perypheral HCs in the health district of Tougan. In this particular context, this intervention could provide an alternative solution to the shortage of human resources for health in rural areas.

## Introduction

Selon l'Organisation mondiale de la santé (OMS) 303 000 femmes sont décédées dans le monde en 2015 en raison de problèmes liés à la grossesse ou à l'accouchement. Environ 99% de ces décès maternels se sont produits dans les pays en développement dont plus de la moitié en Afrique sub-saharienne (57%) [1-3]. Il existe une pénurie de sages-femmes (SF) pour gérer les 15% de grossesses et des accouchements qui s'accompagnent de complications obstétricales. Aussi plus de trois quart des décès pourraient être évités chaque année si l'on développait la profession de sage-femme dans les pays en développement [4, 5]. Au Burkina Faso, selon l'Enquête démographique et de santé (EDS) réalisée en 2010, une femme meurt suite à une complication liée à la grossesse toutes les cinq heures [6]. Le ratio de mortalité maternelle était de 341 pour 100 000 naissances en 2010; aussi les causes obstétricales directes et évitables sont responsables d'environ 80% de ces décès (hémorragies, infections et dystocies etc.) [6, 7]. Il a été montré que l'assistance qualifiée à l'accouchement réduit le risque de mortalité maternelle [8]. Graham et al ont calculé que l'assistance qualifiée à l'accouchement pouvait prévenir entre 16% à 33% des décès maternels [9]. Mais pour certains auteurs, cette relation entre l'assistance qualifiée à l'accouchement et décès maternels n'existe pas dans les pays où le niveau de la mortalité maternelle est intermédiaire ou élevé [10]. La notion de personnel qualifié est très variable. Pour l'OMS, le personnel qualifié regroupe les médecins, les sages-femmes et les infirmiers [11, 12]. La SF joue un rôle important dans la lutte contre la mortalité maternelle. En effet, c'est elle qui est au premier contact avec la femme enceinte pour la consultation prénatale (CPN) et l'accouchement. Il existait au Burkina une SF pour 13 138 habitants pour une norme OMS d'une (01) SF pour 3000 habitants [7]. Ce ratio était d'une (01) SF pour 18 031 habitants dans le District sanitaire de Tougan (DST) qui a un réseau routier impraticable et des ambulances en mauvais état [13]. Pour combler ce déficit en SF et faire face aux difficultés d'évacuation sanitaire des parturientes présentant des complications obstétricales dans le DST au Burkina Faso, il a été conçu en 2013 une stratégie décentralisée de l'offre des soins obstétricaux d'urgence basée sur des interventions ponctuelles de prise en charge des complications obstétricales dans les centres de santé (CS) en milieu rural qui n'avaient pas de SF. Ces missions de prise en charge des complications obtétricales ont été réalisées par des SF appélées SF de zone. La présente étude a eu pour objectif de décrire cette expérience et d'analyser ses résultats après trois (03) ans de mise en œuvre.

## Méthodes

**Cadre de l'étude**: L'étude a été réalisée dans le DST, zone de mise en œuvre de l'intervention. Il est situé à 230 km au nord-ouest de Ouagadougou (capitale du Burkina Faso); en 2015, il comptait 273 392 habitants (dont 60 366 femmes en âge de procréer) desservis par un hôpital de district (HD) appelé centre médical avec antenne chirurgicale (CMA) et 39 centres de santé (CS) périphériques (appelés Centre de santé et de promotion sociale-CSPS) répartis sur une superficie de 5 658 km^2^. Le réseau routier qui joint l'HD aux différents CS est constitué à majorité de pistes qui sont impraticables pendant les trois à cinq mois de période pluvieuse de l'année. La distance moyenne qui sépare l'HD des CS est de 36 km en moyenne (avec des extrêmes de 5 à 82 km), correspondant à environ six heures de route (en aller-retour) en saison sèche. Six (06) CS sont inaccessibles en saison pluvieuse. Les CS du district sont couverts par un système de flotte téléphonique mobile payé par le ministère de la santé. Le DST a enregistré dix (10) décès maternels en 2010, huit (8) en 2011, treize (13) en 2012, sept (07) en 2013, neuf (09) en 2014 et deux (02) en 2015 [13, 14].

**Figure 1:**
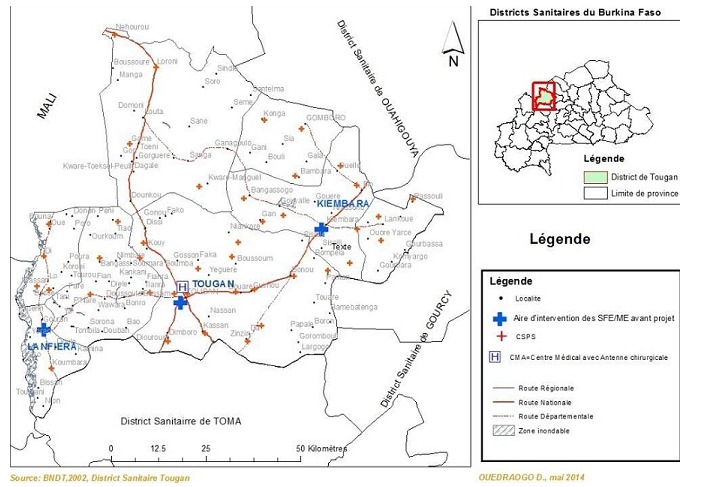
Lieu d'exercice des SF dans les CS du district sanitaire de Tougan (Burkina Faso) avant l’intervention (avant 2013)

**L'intervention Pratique d'offre des soins obstétricaux avant l'intervention**: Avant l'intervention, outre l'hôpital de district, seuls trois CS disposaient d'une SF (Figure 1). Ces SF travaillaient uniquement dans leurs postes d'affectation. Dans les autres CS, les soins obstétricaux étaient offerts par des auxiliaires de soins obstétricaux (accoucheuses auxiliaires) ou par des infirmiers qui ne sont pas suffisamment compétents pour prendre en charge les problèmes obstétricaux graves ou majeurs. Devant une complication obstétricale quelconque, la parturiente était évacuée du CS à l'hôpital de district. Il existait une insuffisance d'offre de soins qualifiés ainsi qu'une rupture de la continuité des soins entre les CS et l'hôpital de district lors des évacuations du fait de l'absence d'une escorte médicale. L'évacuation était assurée par seulement deux ambulances dont la disponibilité était estimée à 56% du fait des pannes récurrentes, du nombre de CS à couvrir et du mauvais réseau routier.

**Figure 2:**
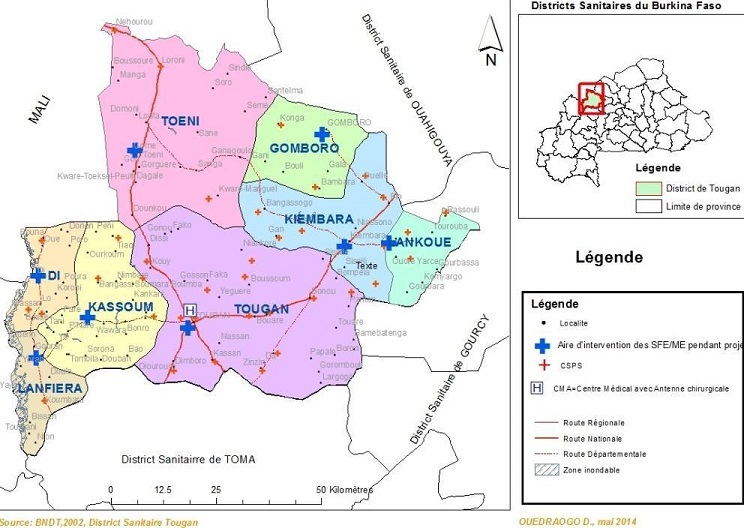
Position géographique et zones de responsabilité des SF de zone pendant l’intervention dans le district sanitaire de Tougan (Burkina Faso)

**Nouvelle organisation du district**: L'intervention a débuté en mai 2013. Elle a consisté d'abord à l'affectation de SF dans cinq (05) autres CS du district. Cela a permis de passer à huit (08) CS disposant d'une SF. Ensuite, le district a été découpé en huit zones sanitaires: Toéni, Kiembara, Kassoum, Lanfièra, Gomboro, Lankoué, Di et Tougan (Figure 2). Le critère principal de découpage du district en zone sanitaire était la distance qui sépare les CS des SF de zone. Chacune de ces SF, responsable de la maternité de son CS et couvrant les autres maternités des CS de sa zone sanitaire, a été nommée SF de zone (Figure 2). La sage-femme de zone (femme ou homme) était une sage-femme qui était affectée dans un CS périphérique et qui intervenait ponctuellement dans un ensemble de CS relevant d'une zone de responsabilité qui lui a été confié. Le nombre de SF de zone ainsi que le nombre de zones sanitaires sont passés à 10 en 2014 à 12 en 2015.

**Responsabilité et attribution des SF de zone**: La gestion des complications obstétricales d'une zone sanitaire donnée regroupant des CS dépourvus de SF a été placée sous la responsabilité d'une SF de zone. Elles apportaient un appui technique sur site en soins obstétricaux d'urgence (SOU) aux auxiliaires et aux infirmiers des CS pour la prise en charge des complications obstétricales en fonction des besoins. Les SF de zone apportaient également un appui à l'équipe cadre du district pour la supervision du personnel des CS ainsi que le suivi des tableaux de bord des indicateurs de la santé de la reproduction des CS de leur zone de responsabilité.

**Figure 3:**
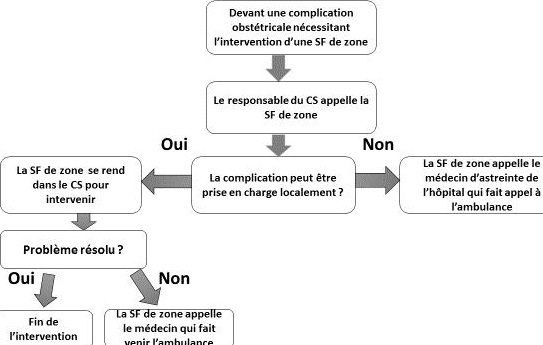
Schéma de l’intervention des SF de zone pour la prise en charge des complications obstétricales dans le district sanitaire de Tougan (Burkina Faso) de 2013 à 2015

**Processus d'intervention des SF de zone dans les CS**: Devant une complication obstétricale survenant dans un CS dépourvu de SF et nécessitant l'intervention d'une SF de zone, le responsable du CS faisait appel immédiatement à la SF de zone à l'aide du téléphone du CS. La SF de zone s'y rendait en motocyclette et prenait en charge la parturiente selon le processus schématisé sur la Figure 3. La SF de zone avertit le médecin d'astreinte de l'HD avant d'effectuer sa mission. Face à certaines complications nécessitant une intervention chirurgicale immédiate, l'ambulance de l'HD était directement sollicitée. Lorsqu'il y avait une non résolution locale de la complication obstétricale par la SF de zone, la patiente était secondairement évacuée à l'HD. Des protocoles de prise en charge des complications obstétricales et des trousses d'urgence ont été mis à la disposition de chaque CS afin de faciliter la mise en œuvre de la stratégie.

**Coût opérationnell:** En termes de coûts opérationnels, les frais de déplacement pour une intervention ont été fixés à 4.25 $ USD pour la restauration de la SF de zone et à 0.07 $ USD le kilomètre parcouru pour le carburant. Ces frais étaient payés par le comité de gestion du CS (COGES) où la complication était survenue.

**Suivi de l'intervention**: Le suivi de cette intervention était assuré par l'Equipe cadre du district (ECD) à travers les sorties de supervisions trimestrielles et la tenue des réunions bilans semestrielles des activités de santé de la reproduction. Au cours de ce suivi les interventions des SF sont examinées et des corrections sont apportées. Des formations continues sur les SOU et la stratégie de décentralisation de l'offre des soins obstétricaux d'urgence ont été organisées au profit des SF de zones.

**Type et période d'étude**: Il s'est agi d'une étude intervention de type transversal analytique. Elle s'est déroulée du 1^er^ Mai 2013 (début de la mise en œuvre de la stratégie) au 31 Décembre 2015 (date d'évaluation de la stratégie).

**Echantillon et échantillonnage**: Les données de toutes les parturientes ayant présenté une complication obstétricale au cours de la mise en œuvre de la stratégie (Mai 2013 à Décembre 2015) et ayant bénéficié de l'intervention des SFE de zone ont été incluses et analysées.

**Collecte des données**: Les données ont été collectées régulièrement au cours de l'intervention. Aussi pour les besoins de l'évaluation, une collecte supplémentaire de données basée sur la méthode de la revue documentaire a été réalisée par quatre (4) enquêteurs préalablement formés du 5 au 20 Janvier 2016 à l'aide d'une grille d'analyse du contenu (grille de collecte) dans les 39 CS du district sanitaire de Tougan. La collecte a consisté essentiellement à une revue des registres d'accouchement des CS et des registres d'intervention des SF de zone.

**Variables d'étude** : La variable « résultat de l'intervention » et son association avec 7 variables indépendantes ont été analysées (Tableau 1 et Tableau 2). Pour le résultat de l'intervention, il a été pris en compte deux modalités (oui ou non): le oui correspondant à la résolution de la complication obstétricale dans le CS d'intervention et le non correspondant à une référence secondaire à l'hôpital de district. Pour les autres variables testées, elles étaient (Tableau 1 et Tableau 2): l'âge, la gestité (nombre de grossesse), la parité, la distance parcourue en km, le délai d'intervention des SF de zone (qui est écoulé à partir de l'appel du CS et le début d'intervention de la SF), l'expérience professionnelle en mois de la SF de zone, le coût des interventions (collecté en CFA et converti en dollar USD: taux d'échange: 1 dollar = 595,743 francs CFA), les complications obstétricales prises en charge (selon le diagnostic posé à partir des signes cliniques et noté dans les registres d'accouchement par les SF de zone) telles les hémorragies (regroupant les hémorragies du troisième trimestre de la grossesse, ainsi que les hémorragies de la délivrance et le placenta prævia), les dystocies (regroupant les dystocies dynamiques et celles mécaniques), les toxémies gravidiques (regroupant les pré-éclampsies, les éclampsies et les hypertensions sur grossesse), les avortements (interruption de la grossesse quelle qu'en soit la cause avant que le fœtus soit apte à la vie extra-utérine c'est-à-dire avant le terme de 22 semaines d'aménorrhée) et les autres complications (infections, cardiopathies, diabètes, drépanocytoses, traumatisme, asthme).

**Tableau 1:** Profil des parturientes prises en charge par les SF de zone et caractéristiques des interventions de mai 2013 à décembre 2015 dans le district sanitaire de Tougan (Burkina Faso)

Variables	n	%
**Age (année)**		
16-19	63	15,1
20-34	300	72,1
35-44	53	12,1
**Gestité**		
0-1	103	24,8
2-4	208	50,0
5 et plus	105	25,2
**Parité**		
0-1	164	39,4
2-4	182	43,8
5 et plus	70	16,8
**Distances parcourues (km)**		
˂15	138	33,2
≥ 15	278	66,8
**Délai écoulé (minutes)**		
˂30	351	84,4
≥30	65	15,6
**Expérience professionnelle des SF (mois)**		
≤12 >12	53 362	12,8 87,2

**Tableau 2:** Relation entre le succès des interventions réalisées par les SF de zone du district sanitaire de Tougan (Burkina Faso) de mai 2013 à décembre 2015 avec les autres variables

Variables	% succès	OR (IC95%)	P-valeur
**Pathologies**			
Hémorragies (110)	80.9	4,50 (1.96-10.4)	˂0.001
Dystocies (211)	84.8	5,94 (2.73-12.96)	
Avortements (41)	80.5	4,38 (1.56-12.29)	
Toxémies (22)	19.0	0,31 (0.10-1.05)	
Autres (33)	48.5	1	
**Age (en mois)**			0,23
16-19 (63)	79.4	1,81 (0.78-4.27)	
20-34 (300)	78.3	1,70 (0.88-3.21)	
35-44 (53)	67.9	1	
**Gestité**			
0-1 (103)	75.7	1,19 (0.64-2.23)	0.27
2-4 (208)	80.3	1,55 (0.90-2.69)	
5 et plus (105)	72.4	1	
**Parité**			
0-1 (164)	75.6	0.85 (0.43-1.66)	0.83
2-4 (182)	78.0	0.97 (0.50-1.89)	
5 et plus (70)	78,6	1	
**Distances parcourues (km)**			
˂15 (138)	73.9	0,76 (0.47-1.23)	0.27
≥ 15 (278)	78.8	1	
**Délai écoulé**			
˂30 (n=351)	77.5	1,12 (0.61-2.08)	0.71
≥30 (n=65)	75.4	1	
**Expérience professionnelle**			
> 12 mois (n=362)	77.3	0,99 (0.50-1.99)	0.998
≤ 12 mois (n=53)	77.4	1	

**Considérations éthiques**: Les données ont été collectées de façon anonyme et routinière. Elle a été faite à l'aide d'un numéro d'identification unique de sorte que l'identification des parturientes ne contienne aucune donnée permettant de les reconnaître. Les informations ont été conservées dans des lieux sécurisés et n'étaient pas accessibles qu'aux personnels de l'étude et à l'ECD.

**Analyse statistique**: Pour la description de notre échantillon, les variables quantitatives ayant une distribution symétrique ont été exprimées par la moyenne et sa déviation standard. Les variables quantitatives ayant une distribution asymétrique ont été représentées par la médiane et ses percentiles (P25 et P75). Les variables qualitatives ont été présentées sous forme de nombre et de proportions. Pour la comparaison des proportions, le test Khi deux (Chi^2^) a été utilisé; des rapports de cotes (OR) des succès des interventions des SFE de zone et leurs intervalles de confiance à 95% ont été calculés. Les analyses ont été effectuées à l'aide des logiciels EPIINFO version 3.5.3 et Open Epi.

## Résultats

**Profil des parturientes prises en charge par les SF de zone**: Le Tableau 1 présente le profil des patientes qui ont bénéficié des interventions des douze SF de zone. Au total, 416 femmes ont été prises en charge du mois de mai 2013 à décembre 2015 (2 ans 7 mois) soit en moyenne treize interventions par mois pour l'ensemble du district et une intervention par SF de zone par mois. L'âge moyen des parturientes était de 26.4 ans (déviation standard = 6.5) avec des extrêmes de 16 ans et de 44 ans (Tableau 1). Un quart de ces parturientes avaient au moins cinq grossesses tandis que 39.4 % d'entre elles étaient nullipares ou primipares (Tableau 1).

**Complications obstétricales prises en charge par les SF**: Les dystocies (osseuses ou dynamiques) représentaient la moitié (50.7%) des complications obstétricales prises en charge par les SF de zone de ces complications suivies des hémorragies (26.4%), des avortements (10%), des pathologies classées autres (8%) (infections, cardiopathies, diabètes, drépanocytoses, traumatisme, asthme) et des toxémies gravidiques (5%).

**Distance parcourue**: Les SF de zone couvraient deux CS en moyenne. La distance médiane parcourue par les SF de zone pour prendre en charge les parturientes était de quinze kilomètres (P25 = 12km, P75 = 16km) avec des extrêmes de cinq kilomètres et de 40 kilomètres (Tableau 1). Aussi 66,8% (IC95%= 62.0-71.3) des distances parcourues par les SF étaient supérieures ou égales à quinze kilomètres.

**Délai d'intervention des SF de zone**: Le délai moyen d'intervention des SF de zone après un appel d'un CS était de 21.1 minutes (déviation standard = 7.1 minutes) avec des extrêmes de dix minutes et de 60 minutes. Près de 85% des interventions ont été réalisées dans un délai de moins de 30 minutes (Tableau 1).

**Expérience professionnelle des SF de zone**: L'expérience professionnelle des SF estimée en nombre de mois au moment des interventions montrait une médiane de 21 mois (P25 = 14 mois, P75 = 33 mois) avec des extrêmes de six mois et de 58 mois. Environ 87% (IC95% = 83.5-90.2) des complications obstétricales ont été prises en charge par des SF qui avaient plus de 12 mois d'expérience professionnelle (Tableau 1).

**Résultats des interventions des sages-femmes**: Les SF de zone ont réalisé 416 missions de prise en charge des complications obstétricales en 2 ans 7 mois (178 interventions en 2013; 136 en 2014 et 102 en 2015). Ce chiffre correspond à 14% (IC95% = 1.3%-1.5%) des accouchements réalisés dans le district en trois ans soit un accouchement sur 74. Sur ces 416 interventions réalisées, 321 ont abouti à la résolution de la complication obstétricale localement, soit une proportion de 77.2%. Ce résultat variait selon le type de complication prise en charge. Il était de 80.9% pour les hémorragies, 84.8% pour les dystocies, 80.5% pour les avortements, 19.0% pour les toxémies gravidiques et 48.5% pour les pathologies classées autres. Quatre-vingt-quatorze patientes (22.8%) ont été référées secondairement à l'hôpital de district après l'intervention des SF de zone. Un décès maternel a été enregistré dans le CS de Débé en mai 2014. Ce CS était situé à 5km de celui qui abritait la SF de zone qui le desservait. Il s'agissait d'une patiente de 32 ans qui avait présenté une hémorragie de la délivrance après un accouchement à domicile. Le décès est survenu dans un tableau d'état de choc avant que la SF qui est arrivée 10 minutes après l'appel de l'accoucheuse auxiliaire ne puisse intervenir.

**Coût des interventions** : Les interventions des SF de zone ont coûté au total 2 572.64 $ USD, soit une moyenne de 6.18 $ USD par intervention et par parturiente avec des extrêmes de 5.16 $ USD et 9.98 $ USD et une déviation standard de 0.72 $ USD. Le financement des interventions a été entièrement assuré par la communauté sous forme de contribution indirecte (ressources des CS provenant de la vente des médicaments ou de la tarification des actes).

**Relation entre le succès des interventions des SF et les autres variables**: Comme le montre le Tableau 2, le type de complications prises en charge était la seule variable déterminant le résultat de l'intervention des SF de zone. Il existait une association statistiquement significative entre le succès des interventions des SF de zone et le type de pathologie prise en charge (OR = 4.50 IC95% = 1.96-10.4 et 5.94, IC95% = 2.73-12.96. p < 0,001). Ainsi plus de 80% des dystocies, des avortements et des hémorragies ont été pris en charge avec succès. Les toxémies gravidiques et les autres pathologies (infections sur grossesse, prolapsus génital sur grossesse, vomissements gravidiques, cardiopathies sur grossesse, anémie sur grossesse, procidence du cordon et utérus cicatriciel) ont été prises en charge avec succès dans respectivement 19% et 48.5%. Par contre, il n'existait pas d'association entre les autres variables (l'âge des parturientes, leurs nombres de grossesses, leurs parités, la distance, l'expérience professionnelle des SF, le délai d'intervention) et le succès des interventions (Tableau 2).

## Discussion

La présente étude a eu pour objectif de décrire l'apport des SF de zone dans la mise en œuvre de la stratégie de décentralisation de l'offre des soins obstétricaux d'urgence du district sanitaire de Tougan. Il s'est agi d'une stratégie basée sur des interventions ponctuelles de prise en charge des complications obstétricales dans les CS péripheriques du district. Des résultats, il est ressorti que la stratégie a permis de prendre en charge 416 femmes en 31 mois dans leur centre de santé de base avec un taux de succès de 77.2%. Aussi, ces parturientes qui présentaient des complications obstétricales ont été prises en charge par les SF de zone avec un délai d'intervention moyen de 21 minutes correspondant à une distance médiane de 15Km parcourus. Par ailleurs, les résultats de l'intervention étaient fonction de la pathologie prise en charge. Nonobstant ces résultats, il a pu être relevé des limites relatives à la présente étude utilisant la revue même si la collecte était routinière. Du reste, la variable délai d'intervention des SF de zone reste une estimation et non une mesure précise collectée au moment de l'intervention. Aussi, la présente étude se focalise sur la santé maternelle et ne prend pas en compte celle du nouveau-né. En outre, il n'a pas été recueilli l'avis des SF de zone ni évaluer s'il y a eu des aspects de rétention des parturientes (ni leurs conséquences) du fait de la prise en charge par les SF de zone. En plus, il n'a pas été possible d'apprécier le statut initial de dispensation des Soins obstétricaux et néonataux d'urgence base (SONUB) des CS avant cette intervention. Elle n'a pas permis non plus de préciser si ces CS acquièrent ce statut par le biais de cette intervention. Si le seul cas de décès signalé présage que cela n'a pas eu d'impact négatif de l'intervention, il est à noter que l'impact de cette intervention sur la réduction de la mortalité maternelle et néonatale n'a pas été évalué. L'offre des soins obstétricaux d'urgence décrite ici a eu pour objectif d'apporter une réponse au déficit en SF dans les CS ruraux du district sanitaire de Tougan ainsi qu'aux difficultés d'évacuation des patientes présentant des complications obstétricales vers l'hôpital de district. En effet, selon l'analyse de la situation sanitaire nationale réalisée lors de l'élaboration du Plan National de Développement Sanitaire (PNDS) 2011-2020 du Burkina Faso, le déficit en SF restait important dans les CS. En effet il existe une SF pour 13 138 habitants pour une norme OMS d'une SF pour 3 000 habitants [7]. Ce ratio est d'une SF pour 18 031 habitants dans le district sanitaire de Tougan qui a un réseau routier impraticable et des ambulances en mauvais état [13].

Par ailleurs, selon le rapport sur l'analyse initiale de la situation de la santé maternelle néonatale et infantile réalisée dans le cadre de l'évaluation des fonds français Muskoka au Burkina Faso en 2012, seulement 14% des accouchements étaient assistés et/ou effectués par les SF [15]. Cette situation interpelle d'autant plus que selon le fonds des nations unies pour la population (UNFPA), le nombre de décès maternels est plus élevé dans les pays où les femmes ont le moins de chance de bénéficier de soins offerts par des professionnels qualifiés comme une SF ou un médecin [2, 3]. Dans ce contexte singulier, la réduction du troisième retard des soins obstétricaux (absence de personnels qualifiés, référencement tardif ou difficile vers le niveau de soins supérieur) pourrait trouver une solution dans cette stratégie de décentralisation de l'offre des soins obstétricaux d'urgence et contribuer à réduire la mortalité maternelle [16,17]. En permettant aux SF de zone d'intervenir sur l'ensemble des CS du district, cette stratégie permet au système local de soins de mieux répondre aux urgences obstétricales comme le recommande le fonds des nations unies pour la population [18, 19]. Les SF de zone ont pris en charge 416 urgences obstétricales correspondant à un accouchement sur 74 (13.4‰ ) accouchements réalisés dans le district en 3 ans. Selon l'UNFPA, une (01) femme africaine sur 39 (25‰) court le risque de mourir de complications liées à la grossesse [18]. L'écart entre les deux chiffres pourrait provenir du fait que le nombre des urgences obstétricales rapporté dans notre étude ne prend pas en compte les complications obstétricales des 12 CS qui abritaient les SF de zone, ni celles qui surviennent à l'hôpital de district chez des parturientes qui s'y rendaient directement. Les complications obstétricales de ces CS sont directement prises en charge par ces SF. Du reste, Bouchra et al. rapportaient une proportion de near miss de 12‰ naissances vivantes en milieu rural au Maroc en 2014 [20]. Les SF de zone ont effectué environ une (01) intervention par mois. Ce nombre moyen paraît convenable et rassure sur la charge de travail supplémentaire éventuelle que cette stratégie pourrait engendrer. Toutefois, une mission par mois pourrait ne pas suffire pour maintenir une compétence suffisante de ces SF de zone. Néanmoins, l'on peut espérer que leur implication dans les activités de l'ECD contribue tant à leur motivation qu'au maintien de leurs compétences. D'autre part, chaque intervention des SF de zone constitue une réelle occasion de formation sur site pour les infirmiers et les auxiliaires qui sollicitent leurs appuis. La répétition de ces occasions d'apprentissage du personnel de santé par les interventions des SF de zone pourrait contribuer à réduire dans le long terme, le nombre de leurs sollicitations. Cela pourrait expliquer en partie la réduction du nombre des interventions durant ces trois ans de mise en œuvre de cette stratégie (178 interventions en 2013; 136 en 2014 et 102 en 2015). Cependant il est à craindre également qu'une (01) mission par mois pourrait ne pas suffire pour ce renforcement des compétences de ce personnel qui sollicite les appuis.

L'âge moyen des parturientes était de 26.4 ans avec des extrêmes de 16 à 44. Cette tranche d'âge correspond à l'âge de procréation des femmes documenté dans la littérature [9, 10]. Bouchra et al. au Maroc rapportaient un âge moyen 29.2 ans chez les nears-miss [21]. Un quart des parturientes avaient eu au moins 5 grossesses tandis que 39.4% d'entre elles étaient nullipares ou primipares. Tarekegn et coll. rapportaient en 2014, que 38.5% de parturientes avaient une parité de 5 enfants au moins en Ethiopie [5]. Dans la présente étude, les dystocies (50.7%) et les hémorragies (26.4%) étaient les pathologies les plus fréquentes suivies des avortements et des toxémies gravidiques. Bouchra et al. rapportaient plutôt 45% de toxémies gravidiques et 39% d'hémorragies chez les nears-miss au Maroc [20]. La prédominance des dystocies dans notre contexte pourrait être due au fait que contrairement aux SF, les accoucheuses auxiliaires et les infirmiers ne sont pas autorisés à utiliser l'ocytocine pour la direction du travail d'accouchement. Les dystocies dynamiques qui étaient autrefois évacuées à l'hôpital de district sont maintenant prises en charge par les SF de zone. Sinon, selon le rapport de l'analyse initiale de la situation de la mortalité maternelle réalisée au Burkina Faso en 2012, les causes obstétricales directes des décès maternels étaient les hémorragies, les infections et les dystocies [15]. Les SF de zone ont réussi à résoudre localement le problème pour lequel elles ont été appelées dans plus de 77% des cas. Le taux de succès était d'autant plus élevé qu'il s'agissait de complications de type hémorragique ou de dystocique. Il était plus faible quand il s'agissait d'une éclampsie ou d'une pré-éclampsie sévère. En effet, les CS périphériques du district sanitaire de Tougan n'étaient pas autorisés à utiliser le Sulfate de magnésium qui est un médicament recommandé pour la prise en charge des éclampsies et des pré-éclampsies sévères. Les cas d'éclampsies étaient stabilisés puis évacués à l'hôpital de référence. Cela corrobore les résultats de l'enquête nationale sur la disponibilité des médicaments vitaux de santé maternelle réalisée au Burkina Faso en 2012 qui rapportait une disponibilité de 43.2% des sept médicaments vitaux en santé de la reproduction dont le sulfate de magnésium dans les CS périphériques de la région de la Boucle du Mouhoun (zone de Tougan) [22]. Au regard du fort potentiel d'aggravation (y compris le risque de décès) que comportent les complications obstétricales, le résultat des interventions des SF de zone ne saurait être évalué par le seul critère de la « résolution locale » ou non de la complication obstétricale.

En d'autres termes, un transfert secondaire d'une parturiente à l'hôpital de référence n'est pas forcément synonyme d'échec dès lors que l'intervention permet de stabiliser la patiente, ou de la maintenir en vie avant son évacuation pour une césarienne. Le décès maternel enregistré dans le CS avant l'intervention de la SF pose avec acuité le problème d'accès aux soins obstétricaux dans notre contexte comme le rappelle si bien Somé et al. dans leur travail sur la perception des femmes sur les accouchements à domicile au Burkina Faso. Les accouchements à domicile seraient liés au faible pouvoir de décision des femmes, à la distance entre les CS et les ménages, ainsi qu'aux contraintes financières [23]. Cette stratégie de décentralisation de l'offre des soins obstétricaux d'urgence conçue localement par l'équipe cadre du district sanitaire de Tougan pour suppléer à l'absence de SF dans les CS périphériques a été financée localement par les ressources de la communauté. Cela conforte cette idée de Méda et al. qui disaient: «Dans les pays à faible revenu, le leadership et la vision des ECD peuvent contribuer à améliorer la couverture universelle des soins» [24]. Cependant, si cet effort de l'ECD a le mérite d'étendre l'offre des soins des SF à tous les CS du district, il est à craindre trois risques éventuels: une rétention non justifiée de parturientes qui devraient bénéficier directement d'une césarienne ou d'un autre soin qui ne peut être offert en périphérie, une mobilisation excessive des SF de zone qui seraient finalement absentes de leur propre poste de travail et un problème de sécurité des SF de zone pendant leurs missions. En effet, relatif à la rétention non justifiée de parturientes, l'ECD a distribué des protocoles de prise en charge des urgences obstétricales précisant les conduites à tenir devant chaque symptôme dans les CS. En outre, il a été demandé aux SF d'avertir le médecin de garde de l'hôpital et de lui présenter le cas clinique avant toute intervention afin de lui permettre d'anticiper et de coordonner à distance la gestion du problème. Devant des complications telles qu'une éclampsie, l'ambulance était systématiquement mise en route. Pour l'aspect concernant la mobilisation excessive des SF de zone, cette crainte reste peu justifiée dans la mesure où les SF de zone ne réalisent en moyenne qu'une seule mission par mois et que l'on constate une réduction progressive du nombre de missions au cours des 3 années de mise en œuvre. Cette réduction pourrait être liée à l'augmentation du nombre de SF dans les CS (8 en 2013, 12 à partir de 2014) et à une probable amélioration de la compétence des infirmiers et des accoucheuses auxiliaires des CS qui bénéficient des interventions des SF de zone. Enfin, un des risques reste le problème de sécurité des SF de zone pendant leurs missions, surtout pendant les nuits. Pour minimiser ce risque, il a été demandé aux SF de zone de se faire accompagner par les parents des patientes ou par les agents de santé communautaire pendant leurs missions de nuit. Aussi, il n'a été noté de cas d'accidents. Loin d'être parfaite, l'intervention des SF de zone offre une opportunité ou une alternative au manque de SF dans les CS en milieu rural et contribue à l'offre de qualité des soins obstétricaux d'urgence en permettant la prise en charge des parturientes dans leur milieu d'origine et en réduisant les dépenses des ménages.

## Conclusion

Une bonne organisation ainsi qu'une bonne gestion des ressources humaines disponibles permet d'améliorer significativement les résultats de la prise en charge des complications obstétricales dans le milieu rural. Cette étude a permis de décrire et d'apprécier le travail des SF de zone du district sanitaire de Tougan. Il s'agit d'une stratégie pertinente qui a permis de suppléer au manque de sages-femmes dans les CS du district. Dans ce contexte particulier, cette intervention pourrait apporter une solution alternative au déficit en ressources humaines en milieu rural. Il faudrait cependant continuer à l'affiner et à renforcer les compétences de ce personnel afin de mieux potentialiser leurs interventions et d'apprécier la qualité des soins offerts. Il conviendrait également de réaliser une étude plus approfondie pour évaluer les opinions de ces acteurs de terrain, la perception de la population vis à vis cette stratégie singulière ainsi que son impact sur la réduction de la mortalité maternelle et néonatale du district.

### Etat des connaissances actuelle sur le sujet

- Bien que de nombreux auteurs aient démontré que l'assistance technique qualifiée à l'accouchement pouvait prévenir entre 16% à 33% des décès maternels [9], les pays en développement peinnent à trouver une reponse adéquate aux problèmes de disponibilité des ressources humaines qualifiées et à l'accessibilité géographique des centres de santé ruraux;
- Aussi des approches similaires d'inversion du système de référence des cas de complications obstétricales sont rares dans la littérature en dépit des documents techniques qui recommandent la réduction des trois retards de la prise en charge de ces cas.

### Contribution de notre étude à la connaissance

- Quand les acteurs locaux du système de santé sont bien encadrés et coachés, ils peuvent impulser un réel leadership à leurs collaborateurs pour faire face à la problématique de la lutte contre la mortalité maternelle dans ce contexte de pénurie en sages-femmes et de routes impraticables;
- Une approche appropriée peut engendrer une implication réelle de la communauté dans la résolution de leur propre problème de santé comme l'indique cette étude (paiement des frais de déplacement des sages-femmes de zone);
- Cette étude confirme le rôle de l'assistance technique qualifiée dans la réduction de la mortalité maternelle.

## Conflits d’intérêts

Les auteurs ne déclarent aucun conflit d'intérêts.
